# Synthesis of Selaginpulvilin D by [2 + 2 + 2] Cyclotrimerization—A Second-Generation Approach

**DOI:** 10.1021/acs.joc.5c02709

**Published:** 2026-01-14

**Authors:** Sundaravelu Nallappan, Lukas Rycek

**Affiliations:** Department of Organic Chemistry, Faculty of Science, 37740 Charles University, Hlavova 8, Prague 128 00, Czech Republic

## Abstract

We report a second-generation synthesis of selaginpulvilin D that addresses key limitations of our earlier route. An efficient early-stage [2 + 2 + 2] cyclotrimerization now provides high-yield access to the molecule’s fluorene core. Previously, this step was low-yielding and relied on a difficult-to-prepare aryldiyne intermediate. By introducing the arylalkyne moiety after the cyclotrimerization, the new strategy removes these issues and delivers a more practical, efficient, and modular pathway to selaginpulvilin D.

## Graphical abstract

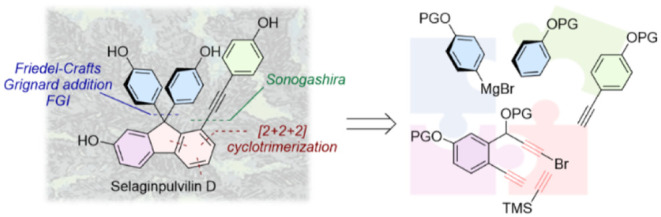

Plants of the genus *Selaginella* are considered living fossils, having persisted on Earth for an estimated 400 million years.^1^ The genus comprises over 700 species, many of which have been used in traditional folk medicine to treat a variety of conditions, including jaundice, gonorrhea, acute hepatitis, asthma, dysmenorrhea, and traumatic injuries.^2,3^ Extracts of these plants have shown various biological activities, such as anticancer, anti-inflammatory, antimicrobial, antioxidant, antiviral, and other *in vitro* and *in vivo* effects.^4–6^ Some species of the genus, particularly *Selaginella pulvinata* and *Selaginella tamariscana*, are rich sources of structurally diverse natural polyphenols—referred to as *selaginellaceae* polyphenols. These include selaginellins,^7^ selagibenzophenones,^8–12^ and selaginpulvilins,^13–15^ among others.^16^ Notably, many of these compounds are unique to these species and have not been identified in any other plants. The structural peculiarity and biological activity of *selaginellaceae* polyphenols have attracted the attention of many researchers, including us. We have been engaged in the development of the synthesis of several natural products, including selagibenzophenones A and C.^17,18^ We also demonstrated that a compound previously isolated and described as selagibenzophenone B was incorrectly elucidated; in fact, the authors had isolated selagibenzophenone A.^17^ We further developed the synthesis of selaginpulvilin X,^19^ selaginpulvilin C, and selaginpulvilin D.^20^ In addition, we developed the synthesis of unnatural derivatives of selagibenzophenone A and B and discovered their selective cytotoxic properties against prostate cancer cell lines.^21–23^

Among the natural products, selaginpulvilins (such as selaginpulvilin D, Figure 1), isolated from *Selaginella pulvinata*, are especially remarkable for two reasons: their structurally unique fluorene-based frameworks, which attract the attention of synthetic chemists, and their promising biological activity, underscoring their potential importance in medicinal chemistry. Selaginpulvilins exhibited strong inhibitory activity against PDE4D2, with IC_50_ values as low as 0.11 μM.^13–15,24^ This potent activity may help explain the healing effect of *Selaginella pulvinata*.

**Figure 1:**
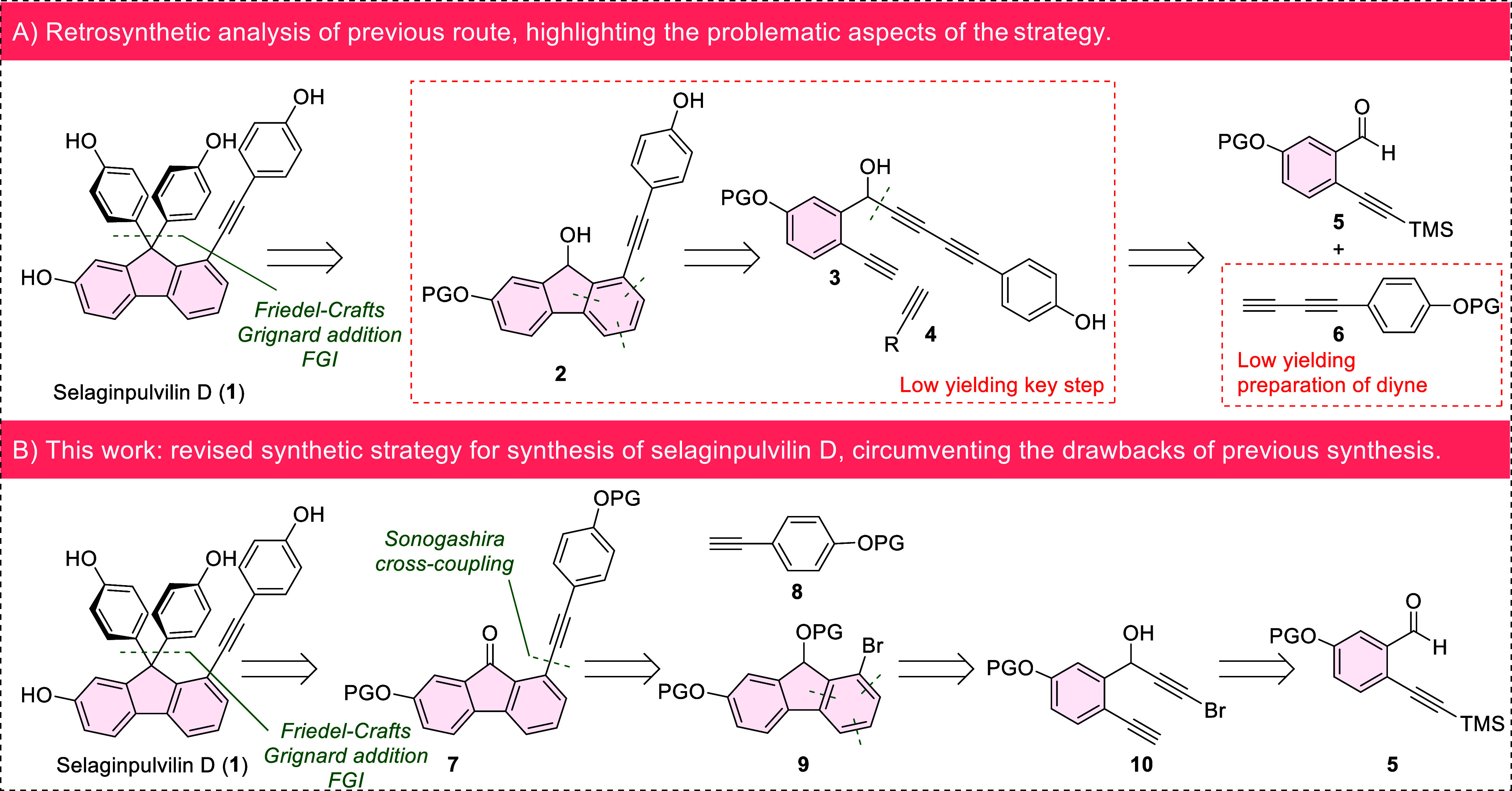
(A) The previously reported synthesis of selaginpulvilin D, based on a partially intramolecular [2 + 2 + 2] cyclotrimerization of triyne **4** and an external alkyne. The key limitations of the synthesis are depicted in the red boxes. (B) The second-generation synthesis of selaginpulvilin D, based on [2 + 2 + 2] cyclotrimerization of bromodiyne **10** and an external alkyne, effectively eliminating the main drawbacks of the initial synthesis.

Several syntheses were developed toward various selaginpulvilins, including strategies based on: (a) a tetradehydro Diels–Alder reaction of an enyne–diyne,^25,26^ (b) a hexadehydro Diels–Alder reaction of a tetrayne,^27^ and (c) sequences comprising cross-coupling reactions and an intramolecular S_E_Ar reaction.^28^ Moreover, we have recently disclosed a new synthesis of selaginpulviline C and D based on [2 + 2 + 2] cyclotrimerization of a substituted 1,3-diyne-yne **3** with alkynes **4** (Figure 1, A).^20^ [2 + 2 + 2] cyclotrimerization is an efficient method for constructing aromatic and heteroaromatic compounds,^29^ using various catalysts such as rhodium,^30^ ruthenium,^31^ cobalt,^32^ nickel,^33^ iron,^34^ or others.^29^ It has been successfully applied in the preparation of various natural products^35^ or molecules with potential applications in material sciences.^36^ The effectiveness of our modular approach in the synthesis of selaginpulvilin D was, however, compromised by a low yield of the key [2 + 2 + 2] cyclotrimerization step, particularly in the case of selaginpulvilin D. The desired fluorenol **2** was obtained in only 36%. Another problem that we encountered was the low-yielding preparation of diyne **6**, which was obtained in three steps with a poor 27% yield.

Herein, we report a revised synthetic strategy for the formal total synthesis of selaginpulvilin D (**1**), based on a [2 + 2 + 2] cyclotrimerization of brominated diyne **10** (Figure 1, B). This approach enables the early construction of the substituted fluorene core in good yield. The arylalkyne moiety is introduced into the molecule after the key [2 + 2 + 2] cyclotrimerization via Sonogashira coupling of the bromide with arylacetylene **8**, allowing us to circumvent the use of diyne **6**. Subsequent transformations of fluorene **9** complete the formal synthesis of selaginpulvilin D while overcoming the main limitation of the previously reported route.

Synthesis began from alkyne **11**,^20^ which was treated with ethynylmagnesium bromide, and the resulting alcohol was, without further purification, protected with a MOM group to afford alkyne **12** in an overall 72% yield. Bromination of the terminal alkyne and basic methanolysis of the TMS group provided the key dialkyne **13** in 89% and 79% yield, respectively (Scheme 1, A).

**Scheme 1:**
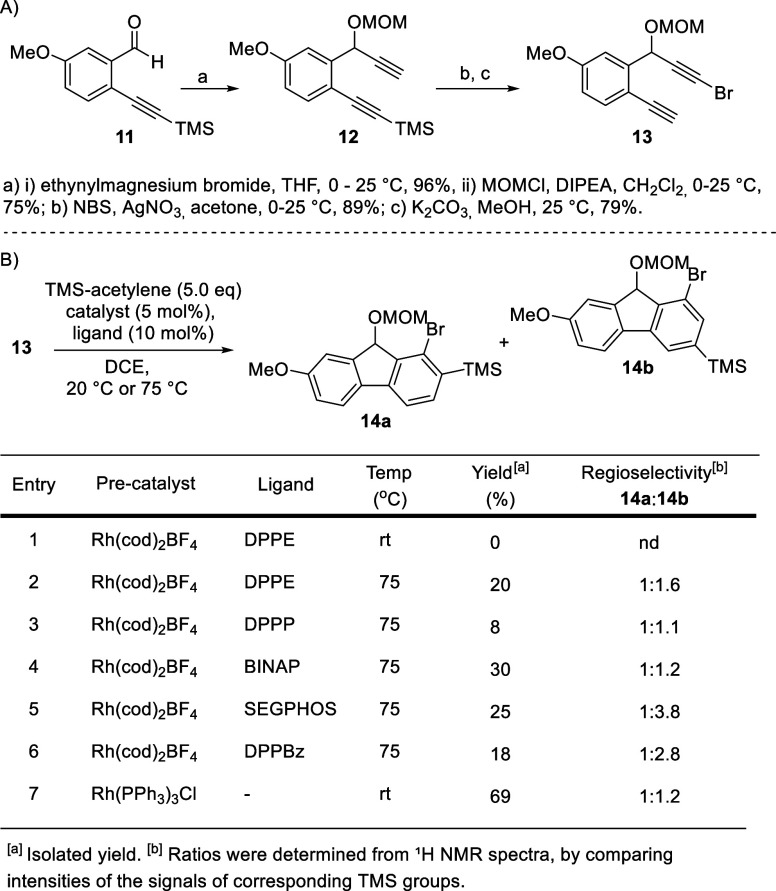
(A) Synthesis of the [2 + 2 + 2] Cyclotrimerization Precursor. (B) Optimization of the Key [2 + 2 + 2] Cyclotrimerization Step

Having obtained diyne **13**, we carried out a [2 + 2 + 2] cyclotrimerization to construct the key structural motif of selaginpulvilin D (**1**), namely, the fluorene core. Based on our previous experience,^36b,37^ we examined catalytic rhodium precatalyst Rh­(COD)_2_BF_4_ in combination with various bidentate ligands, such as DPPE, DPPP, BINAP, SEGPHOS, and DPPBz. However, the reactions provided the desired fluorene in unsatisfactory yields, ranging between 8% and 30% (Scheme 1, Table 1, entries 1–6). We did not observe any significant or only slightly increased selectivity for regioisomer **14b** (up to 1:3.8 for **14a:14b**). To our delight, the yield increased to a good 69% when the reaction was carried out with Wilkinson’s catalyst at room temperature (Scheme 1, Table 1, entry 7). We obtained a mixture of two regioisomers, **14a** and **14b,** in the ratio of 1:1.2 (**14a:14b**). The low selectivity of the transformation does not pose a significant problem for the total synthesis, since both isomers can be converted into the natural product in subsequent steps.

To finalize the synthesis of **1**, the mixture of regioisomers **14a** and **14b** was, in two steps, converted to fluorenone **15** (Scheme 2). First, treatment of **14a** and **14b** with TBAF resulted in the cleavage of the TMS group from the aromatic core and, to our delight, also in the deprotection of the MOM group, followed by spontaneous oxidation to fluorene. Further Sonogashira cross-coupling with 4-methoxyphenylacetylene, catalyzed by XPhosPdG2, furnished the formation of **15**, which was obtained in 67% and 58% yields for the respective steps. Further introduction of two aromatic rings by means of a reaction with 4-methoxyphenylmagnesium bromide and a reaction with anisole led to the formation of the protected selaginpulvilin **16** in 90% and 75% yield, respectively. Deprotection of **16** was described previously; thus, the formal synthesis of the natural product was achieved.

**Scheme 2:**
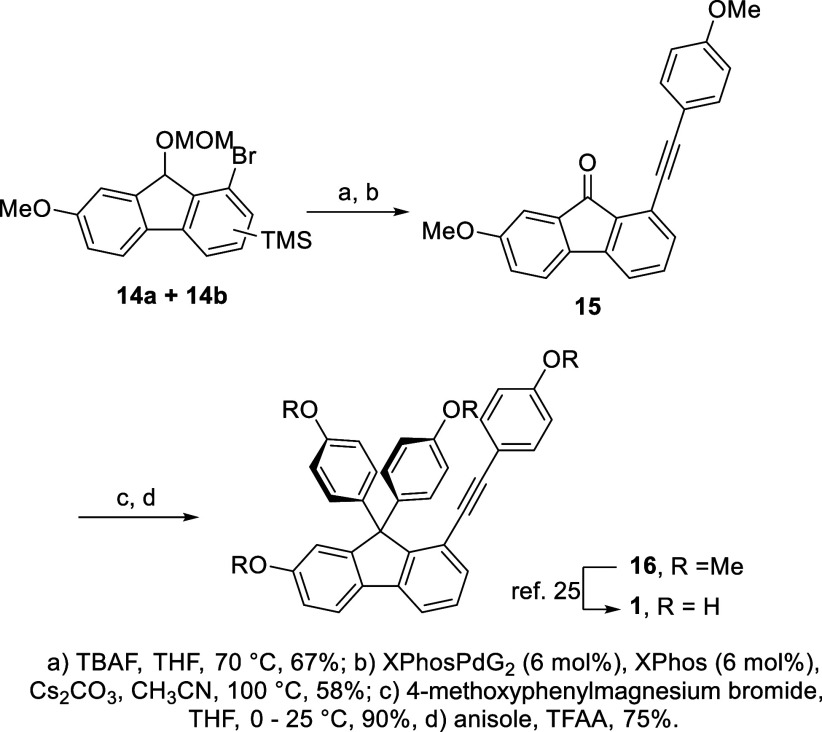
Finalization of the Formal Synthesis of Selaginpulvilin D

In conclusion, we report a modular formal synthesis of selaginpulvilin D (**1**). The key fluorene core of the natural product was constructed via a partially intramolecular [2 + 2 + 2] cyclotrimerization between diyne **13** and trimethylsilyl acetylene, providing the desired fluorenol in 69% yield. This represents a significant improvement over that of our previous synthesis. The fluorenol was obtained as a mixture of two regioisomers; however, this does not affect the total synthesis, as TMS removal from both isomers yields the same product. The formal synthesis was completed by introducing the arylalkynyl moiety via Sonogashira cross-coupling using 4-methoxyphenylacetylene, which overcomes the low-yield issue encountered with aryldiyne **6** in the earlier route. Finally, two aromatic rings were installed at position 2 of the fluorene core to complete the formal total synthesis. Overall, the new strategy provides an efficient and improved route to selaginpulvilin D (**1**), addressing several limitations of our previous synthesis.

## Supplementary Material



## Data Availability

The data underlying this study are available in the published article and its Supporting Information.

## Notes

Experimental procedures, characterization data for all compounds, and copies of NMR spectra (PDF)
