# Supplementary material for: Synthesis of Selaginpulvilin D by [2 + 2 + 2] Cyclotrimerization—A Second-Generation Approach
Source: J Org Chem. 2026 Jan 14;91(4):1860–3. doi: 10.1021/acs.joc.5c02709 (PMC12865770; doi:10.1021/acs.joc.5c02709)
Supplement: Supplementary file 1 [file jo5c02709_si_001.pdf]

Supplementary information

Synthesis of Selaginpulvilin D by [2+2+2] Cyclotrimerization, a Second-Generation Approach.

Sundaravelu Nallappan, and Lukas Rycek*

Department of Organic Chemistry, Faculty of Science, Charles University, Hlavova 8,
128 00 Prague (Czech Republic) Phone: +420 221 95 1981; E-mail: rycekl@natur.cuni.cz

Table of Contents

1. Experimental section 2
2. Protocols of preparations and spectral characterization of compounds....................2
3. Copies of spectra: 8
4. References....................17

1. Experimental section

All used chemicals were bought from OrgChem, Sigma–Aldrich, Fluorochem, Acros Organics, PENTA Chemicals, Alfa Aesar, BLDpharm, Strem Chemicals, Tokyo Chemical Industry, and Lach:ner. For TLC analysis a UV lamp with a wavelength of 254 nm and TLC plates with F254 bare silica from Silicycle were used. For preparative TLC glass backed TLC plates with 60A F254 silica gel from Silicycle were used. For column chromatography, silica gel 60A (0.040–0.063 mm) was used. All NMR spectra were measured on Bruker Avance III and Bruker Avance NEO 400 MHz spectrometers (400 MHz for ^{1}H and 101 MHz for ^{13}C) and Bruker Avance III 600 MHz spectrometer (600 MHz for ^{1}H and 151 MHz for ^{13}C). MS were obtained on a VG-Analytical ZAB SEQ spectrometer. ESI-MS detection was conducted on a Bruker QqTOF compact instrument operated using Compass otofControl 4.0 (Bruker Daltonics, Germany) software. Compass DataAnalysis 4.4 (Build 200.55.2969) (Bruker Daltonics, Germany) software was used for data processing. Artificial intelligence, ChatGPT (OpenAI), was employed as a supplementary tool during the preparation of this article. It was utilized for providing linguistic corrections, stylistic suggestions, and improving clarity in English and for shortening sections and eliminating redundancy. All AI-generated outputs were carefully reviewed, edited, and adapted by the author to ensure accuracy and compliance with academic standards. The use of AI aligns with ethical practices and does not substitute for the author's critical analysis and independent research.

2. Protocols of preparations and spectral characterization of compounds.

1-(5-methoxy-2-((trimethylsilyl)ethynyl)phenyl)prop-2-yn-1-ol (S1):

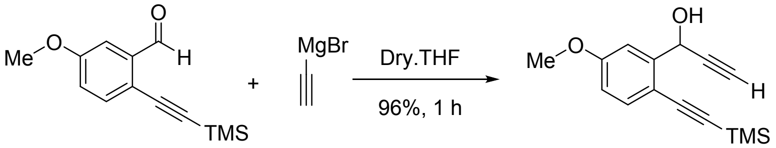

11 **S1**

A solution of ethynyl magnesium bromide (21 mL, 0.5 M in THF) was added in a dropwise manner to a solution of the benzaldehyde **11** in THF at 0 °C. After stirring the reaction in the ice bath for 40 min, it was quenched with saturated NH_4Cl solution. The product was extracted with ethyl acetate (3 × 15 mL). Combined organic phases were dried over Na_2SO_4, then filtered and concentrated, yielding 2.35 g of corresponding secondary benzyl alcohol product (96% yield) in the form of a colorless glassy oil, which was used without further purification in the next step. R_f 0.35 (10% ethyl acetate in hexanes). ^{1}H NMR (400 MHz, $CDCl_3$) δ 7.44 (d, J = 8.5 Hz, 1H), 7.23 (d, J = 2.6 Hz,

1H), 6.83 (dd, J = 8.5, 2.6 Hz, 1H), 5.82 (dd, J = 5.7, 2.3 Hz, 1H), 3.86 (s, 3H), 2.92 – 2.85 (m, 1H), 2.66 (d, J = 2.3 Hz, 1H), 0.28 (s, 9H). 13C{1H} NMR (101 MHz, $CDCl_3$) δ 160.2, 144.0, 134.2, 114.0, 113.2, 112.2, 102.2, 99.1, 82.5, 74.7, 63.2, 55.4, -0.1. HRMS (ESI) m/z: $[M+Na]^+$ Calcd for $C_{15}H_{18}NaO_2Si$: 281.0968; Found: 281.0965.

((4-methoxy-2-(1-(methoxymethoxy)prop-2-yn-1-yl)phenyl)ethynyl)trimethylsilane (12):

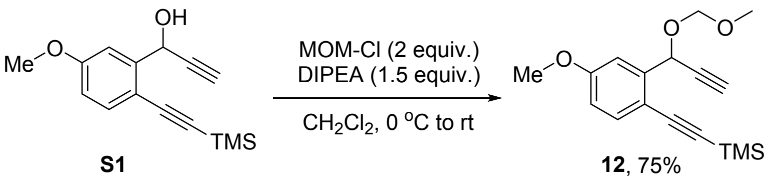

Under an open atmosphere, secondary benzyl alcohol **S1** (2.34 g, 9.06 mmol, 1.0 equiv.,) was dissolved in CH_2Cl_2 solvent and the solution was brought to 0 °C, and the DIPEA (2.37 mL, 13.59 mmol, 1.5 equiv.) was successively added to the reaction, followed by MOM-Cl (13.76 mL, 18.12 mmol, 2.0 equiv.) was added and closed with glass-stopper. The reaction was allowed until the completion of the starting material. Then, the reaction mixture was quenched with water and extracted with ethyl acetate (3×15 mL). And the organic layer was washed with 10% HCl and saturated $NaHCO_3$ solution followed by brine wash (1×15 mL). The organic fraction was dried over anhydrous Na_2SO_4. Removal of solvent and silica gel column chromatography using hexanes and ethyl acetate mixture (95/5) afforded 2.1 g of the title compound (75% yield) in a form of a pale yellow liquid; R_f 0.50 (10% ethyl acetate in hexanes). ^1H NMR (400 MHz, $CDCl_3$) δ 7.43 (d, J = 8.6 Hz, 1H), 7.30 – 7.25 (d, J = 2.7 Hz, 1H), 6.84 (dd, J = 8.6, 2.7 Hz, 1H), 5.88 (d, J = 2.2 Hz, 1H), 5.02 (d, J = 6.7 Hz, 1H), 4.79 – 4.72 (m, 1H), 3.86 (s, 3H), 3.46 (s, 3H), 2.60 (d, J = 2.2 Hz, 1H), 0.28 (s, 9H). 13C{1H} NMR (101 MHz, CDCl3) δ 170.9, 141.9, 134.0, 114.6, 114.3, 112.6, 102.2, 98.0, 94.6, 81.4, 74.9, 65.2, 56.0, 55.4, 0.0. HRMS (ESI) m/z: $[M+Na]^+$ Calcd for $C_{17}H_{22}NaO_3Si$: 325.1230; Found: 325.1227.

((2-(3-bromo-1-(methoxymethoxy)prop-2-yn-1-yl)-4-methoxyphenyl)ethynyl)trimethylsilane (S2):

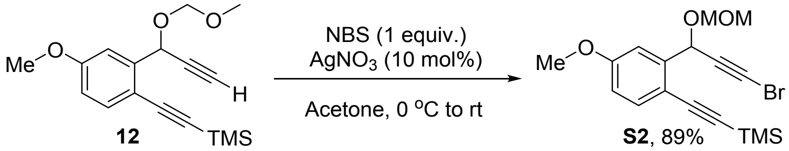

In the round bottom flask, under an argon atmosphere 1-ethynyl-4- methoxybenzen (**12**) (2.1 g, 6.80 mmol, 1.0 equiv.) and freshly recrystallized *N*-bromosuccinimide (1.21 g, 6.80 mmol, 1.0 equiv.) and $AgNO_3$ (0.12 g, 0.68 mmol, 0.1 equiv.) was dissolved in acetone (50 mL). The resulting mixture was stirred for 1 hour at 25 °C, where upon the solvent was evaporated under reduced pressure. The column chromatography of the crude mixture using silica gel and hexanes and ethyl acetate mixture (98:2) provided 2.31 g of the title compound (89% yield) in a form of colorless glassy oil. R_f 0.60 (10% ethyl acetate in hexanes). ^1H NMR (400 MHz, $CDCl_3$) δ 7.43 (d, J = 8.6 Hz, 1H), 7.22 (d, J = 2.7 Hz, 1H), 6.84 (dd, J = 8.6, 2.7 Hz, 1H), 5.88 (s, 1H), 5.01 (d, J = 6.7 Hz,

1H), 4.74 (d, J = 6.7 Hz, 1H), 3.86 (s, 3H), 3.45 (s, 3H), 0.28 (s, 9H). 13C{1H} NMR (101 MHz, CDCl3) δ 160.1, 141.8, 134.0, 114.5, 114.2, 112.6, 102.1, 98.1, 94.6, 77.9, 66.1, 56.0, 55.4, 46.9, 0.0. HRMS (ESI) m/z: [M+Na]+ Calcd for $C_{17}H_{21}BrNaO_3Si$: 403.0335; Found: 403.0336.

2-(3-bromo-1-(methoxymethoxy)prop-2-yn-1-yl)-1-ethynyl-4-methoxybenzene (13):

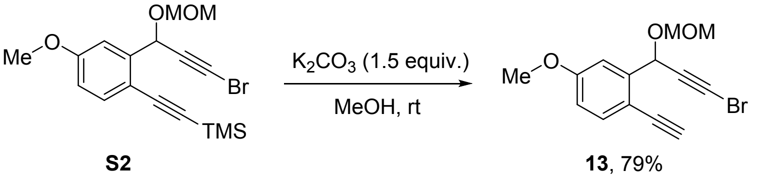

In a round bottom flask was dissolved the starting diyne **S2** (2.00 g, 5.24 mmol, 1.0 equiv.) in MeOH (30 mL) and K_2CO_3 (1.09 g, 7.86 mmol, 1.5 equiv.) was added. The resulting mixture was allowed to stir at 25 °C for 30 minutes, after which ethyl acetate (30 mL) was added. The organic phase was washed with the saturated solution of NH_4Cl, dried over $MgSO_4$, filtered, and volatiles were removed under reduced pressure. The column chromatography of the crude mixture using silica gel and hexanes and ethyl acetate mixture (10/1 to 2/1) provided 1.27 g of the title compound (79% yield) in a form of light-yellow oil. R_f 0.30 (10% ethyl acetate in hexanes); ^{1}H NMR (400 MHz, $CDCl_3$) δ 7.46 (d, J = 8.5 Hz, 1H), 7.24 (d, J = 2.7 Hz, 1H), 6.86 (dd, J = 8.5, 2.7 Hz, 1H), 5.90 (s, 1H), 5.01 (d, J = 6.8 Hz, 1H), 4.72 (d, J = 6.8 Hz, 1H), 3.87 (s, 3H), 3.45 (s, 3H), 3.27 (s, 1H). 13C{1H} NMR (101 MHz, CDCl3) δ 160.4, 141.9, 134.3, 114.7, 113.1, 112.8, 94. 5, 80.9, 80.8, 77.7, 66.0, 56.1, 55.5, 47.2. HRMS (ESI) m/z: [M+Na]+ Calcd for $C_{14}H_{13}BrNaO_3$: 330.9940; Found: 330.9939.

(1-bromo-7-methoxy-9-(methoxymethoxy)-9H-fluoren-2-yl)trimethylsilane (14a) and (1-bromo-7-methoxy-9-(methoxymethoxy)-9H-fluoren-3-yl)trimethylsilane (14b):

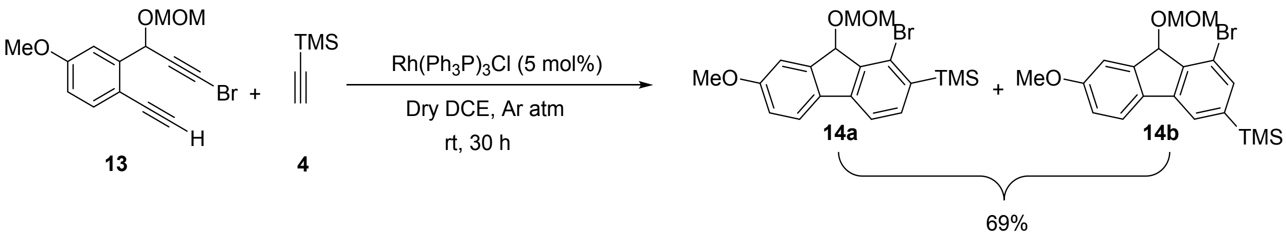

A microwave vial was charged with bromoalkyne **13** (32 mg, 0.10 mmol), and Wilkinson's catalyst (9.4 mg, 0.01 mmol). The atmosphere was exchanged for argon and the mixture was dissolved in dry DCE (2 mL). Alkyne **4** (0.5 mmol) was added, and the reaction mixture was sealed and allowed to stir at RT. The reaction was monitored by TLC, after the completion of the starting material, the mixture was passed through a pad of Celite® and silica gel and the solvent was evaporated under

reduced pressure. Column chromatography of the residue on silica gel yielded the desired product in 69% yield as a yellowish oil. ^{1}H NMR (400 MHz, $CDCl_3$, mixture of regioisomers) δ 7.61 (s, 1H), 7.58 (d, J = 8.3 Hz, 1H), 7.54 (d, J = 8.3 Hz, 1.20H), 7.47 (m, 2.21H), 7.42 (d, J = 7.5 Hz, 1.20H), 7.30 – 7.26 (m, 2.92H, with residual solvent), 6.95 (m, 2.24H), 5.51 (m, 2.21), 5.08 (d, J = 6.9 Hz, 1.18H), 5.05 (d, J = 6.9 Hz, 1H), 4.80 – 4.83 (m, 2.20H), 3.90 (s, 6.88H), 3.48 (s, 3H), 3.47 (s, 3.70) 0.44 (s, 10.89H), 0.33 (s, 9H). 13C{1H} NMR (101 MHz, $CDCl_3$) δ 160.5, 160.3, 144.9, 144.9, 144.8, 143.9, 142.6, 142.2, 142.0, 139.3, 137.4, 134.8, 132.6, 132.5, 128.9, 122.5, 121.2, 121.04, 117.4, 115.2, 115.1, 111.9, 97.5, 97.2, 82.6, 81.8, 56.4, 55.6, -0.1, -1.1. HRMS (ESI) m/z: [M+Na]+ Calcd for $C_{19}H_{23}BrNaO_3Si$: 429.0492; Found: 429.0490.

Characteristic peaks for **14a** isomer: ^{1}H NMR (400 MHz, $CDCl_3$) δ 7.54 (d, J = 8.3 Hz, 1H), 7.44 (d, J = 7.5 Hz, 1H), 0.44 (s, 9H)

Characteristic peaks for **14b** isomer: ^{1}H NMR (400 MHz, $CDCl_3$) δ 7.61 (s, 1H), 7.58 (d, J = 8.3 Hz, 1H), 0.33 (s, 9H)

1-bromo-7-methoxy-9H-fluoren-9-one (S3):

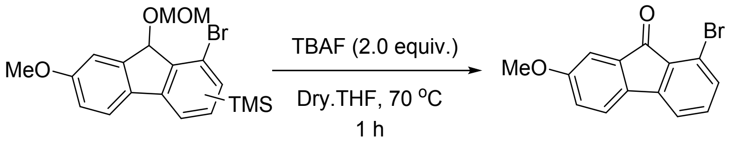

14a + **14b** **S3**, 67%

A mixture of isomers **14a** and **14b** (499 mg, 1.22 mmol) was dissolved in a solution of TBAF in THF (2 equiv.) under argon and heated to 70 °C using oil bath for 1.5 hours. After that, EtOAc (2 mL) was added and the mixture was successively washed with water (10 mL) and brine (10 mL). The combined organic layers were dried over $MgSO_4$, filtered, and volatiles were removed under reduced pressure. The column chromatography of the crude mixture using silica gel and hexanes and ethyl acetate mixture (10/1 to 4/1) provided 237 mg of the title compound (67% yield) in a form of a yellow solid. ^{1}H NMR (400 MHz, $CDCl_3$) δ 7.42 (d, J = 8.3 Hz, 1H), 7.39 – 7.30 (m, 2H), 7.29 – 7.24 (m, 1H), 7.22 (d, J = 2.4 Hz, 1H), 7.02 (dd, J = 8.3, 2.4 Hz, 1H), 3.88 (s, 3H). 13C{1H} NMR (101 MHz, $CDCl_3$) δ 191.0, 161.3, 147.4, 135.7, 135.4, 134.9, 132.9, 131.3, 121.4, 120.6, 120.4, 118.5, 109.3, 55.8. The spectroscopic data is in accordance with the one reported in the literature.[1]

7-methoxy-1-((4-methoxyphenyl)ethynyl)-9H-fluoren-9-one (15):

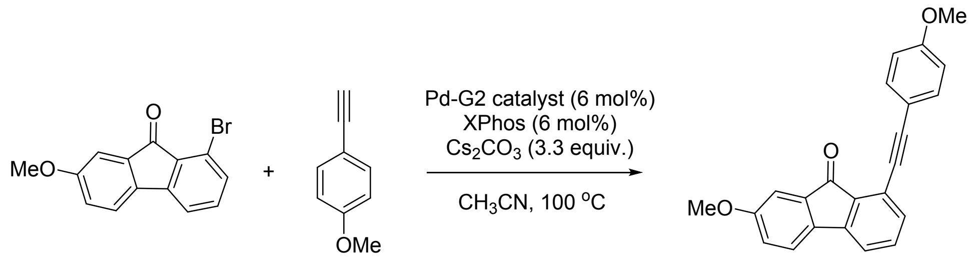

S3 **15**, 58%

1-Bromo-7-methoxy-9H-fluorene **S3** (90 mg, 0.31 mmol, 1.0 equiv), 1-ethynyl-4-methoxybenzene (123 mg, 0.93 mmol, 3 equiv), Pd-XPhos generation 2 pre-catalyst (15 mg, 6 mol %), XPhos (9 mg, 6 mol %), Cs_2CO_3 (333 mg, 1.02 mmol, 3.3 equiv), acetonitrile (4.5 mL) were placed in a 100 mL thick-walled glass pressure tube at 100 °C in an oil bath and stirred for 19 h. The solution was cooled to room temperature, diluted with water and extracted with ethyl acetate (3 x 5 mL). The organic extracts were combined, dried over anhydrous $MgSO_4$, filtered and concentrated under reduced pressure. The column chromatography of the crude mixture using silica gel and hexanes and ethyl acetate mixture (10/1 to 4/1) provided 61 mg of the title compound (58% yield) in a form of an orange solid. ^{1}H NMR (400 MHz, $CDCl_3$) δ 7.68 (d, J = 8.8 Hz, 2H), 7.45 – 7.34 (m, 3H), 7.32 – 7.25 (m, 2H), 7.01 (dd, J = 8.2, 2.5 Hz, 1H), 6.94 (d, J = 8.8 Hz, 2H), 3.88 (s, 3H), 3.87 (s, 3H). 13C{1H} NMR (101 MHz, $CDCl_3$) δ 192.1, 161.3, 160.3, 145.4, 136.2, 135.8, 134.1, 133.9, 133.5, 131.7, 121.7, 121.4, 120.2, 118.8, 115.4, 114.2, 109.2, 96.3, 85.6, 55.8, 55.4. The spectroscopic data is in accordance with the one reported in the literature.[1]

7-methoxy-9-(4-methoxyphenyl)-1-((4-methoxyphenyl)ethynyl)-9H-fluoren-9-ol (S4):

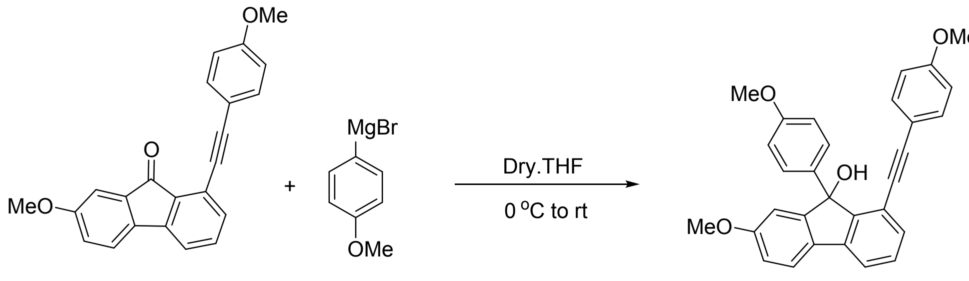

15 **S4**, 90%

To a stirred solution of fluorenone **15** (34 mg, 0.1 mmol, 1.0 equiv) in THF (2 mL) was added 1 M solution of 4-methoxyphenylmagnesium bromide in THF (0.11 mL, 1.1 equiv) at 0 °C (ice-water bath). After 15 min, cold water was added to quench the reaction and extracted with ethyl acetate (3 x 2 mL). The organic layer was washed with brine, dried over anhydrous $MgSO_4$, filtered and concentrated. The crude material was passed through a short silica gel column (eluent: hexane/ethyl acetate 10:1 to 4:1) to yield 41 mg of the title compound (90%) in a form of a white

solid. ^{1}H NMR (400 MHz, $CDCl_3$) δ 7.57 (m, 2H), 7.41 – 7.28 (m, 4H), 7.18 (d, J = 8.8 Hz, 2H), 6.92 – 6.87 (m, 2H), 6.87 – 6.81 (m, 4H), 3.84 (s, 3H), 3.79 (s, 3H), 3.76 (s, 3H). 13C{1H} NMR (101 MHz, $CDCl_3$) δ 160.7, 159.9, 158.9, 152.1, 150.5, 141.0, 135.4, 133.1, 131.1, 130.2, 129.3, 126.6, 121.3, 119.6, 119.1, 115.1, 114.9, 114.0, 113.7, 110.1, 95.5, 85.0, 83.9, 55.6, 55.4, 55.4. The spectroscopic data is in accordance with the one reported in the literature.[1]

7-methoxy-9,9-bis(4-methoxyphenyl)-1-((4-methoxyphenyl)ethynyl)-9H-fluorene (16):

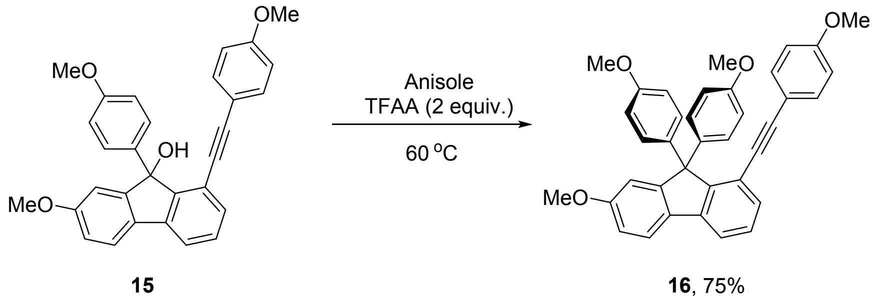

To a stirred solution of alcohol **15** (41 mg, 0.09 mmol) in anisole (1 mL), TFAA (57 mg, 0.27 mmol, 3 equiv.) was added at 5 °C (ice-water bath). The cooling bath was removed and the mixture was allowed to stir at 60 °C in an oil bath. After 30 minutes, cold water (2 mL) and CH_2Cl_2 (5 mL) were added and the organic layer was separated. The aqueous layer was again extracted with 5 mL of CH_2Cl_2. The combined organic layer was dried over anhydrous $MgSO_4$, filtered and concentrated over reduced pressure. The column chromatography of the crude mixture using silica gel and hexanes and ethyl acetate mixture (10/1 to 4/1) provided 36 mg of the title compound (75% yield) in a form of a yellow oil. ^{1}H NMR (400 MHz, $CDCl_3$) δ 7.71 – 7.62 (m, 2H), 7.39 – 7.32 (m, 2H), 7.30 – 7.20 (m, 4H), 7.05 – 6.98 (m, 2H), 6.94 – 6.86 (m, 2H), 6.86 – 6.80 (m, 2H), 6.80 – 6.71 (m, 4H), 3.83 (s, 3H), 3.78 (s, 3H), 3.76 (s, 6H). 13C{1H} NMR (101 MHz, $CDCl_3$) δ 160.4, 159.6, 158.3, 155.6, 151.9, 140.9, 135.0, 132.8, 132.2, 130.99, 130.5, 127.7, 121.2, 120.8, 119.3, 115.7, 114.0, 113.5, 113.2, 111.2, 96.0, 87.7, 65.1, 55.6, 55.4, 55.3.

3. Copies of spectra:

Figure S1 ^{1}H NMR spectra of metal compound **S1** in $CDCl_3$ (400 MHz)

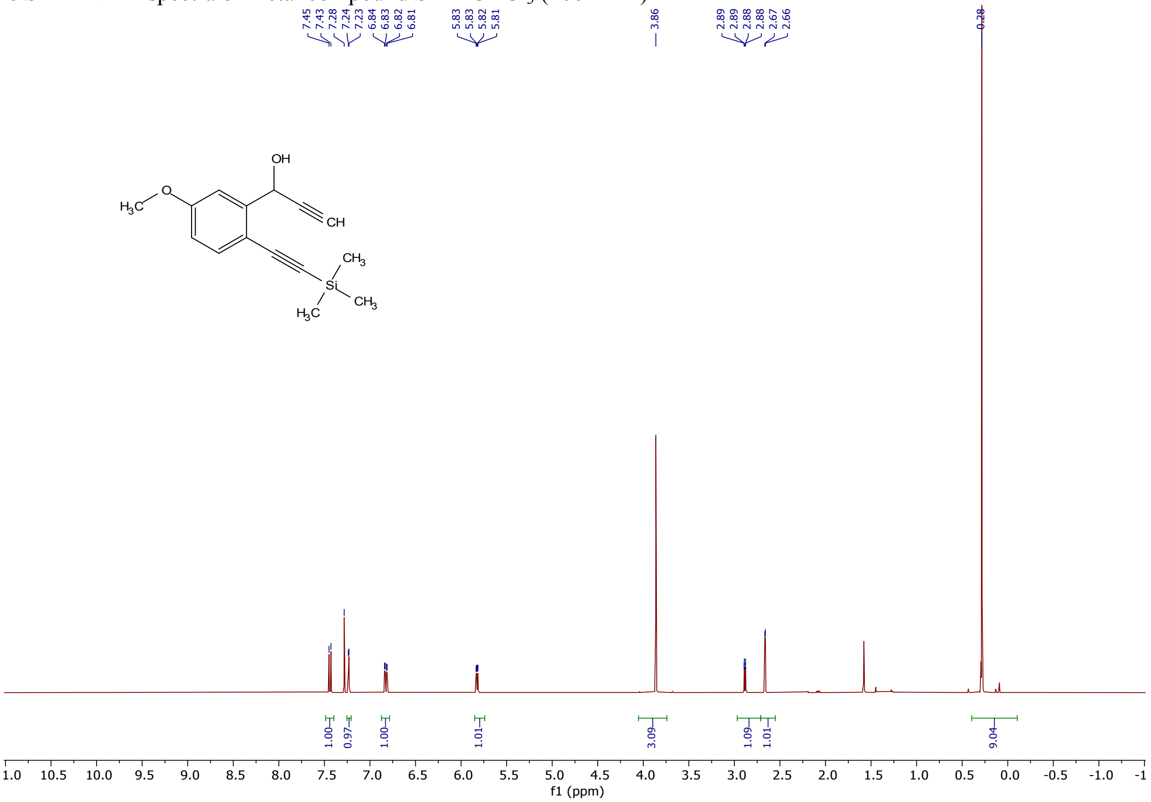

Figure S2 13C{1H} NMR spectra of compound **S1** in $CDCl_3$ (101 MHz)

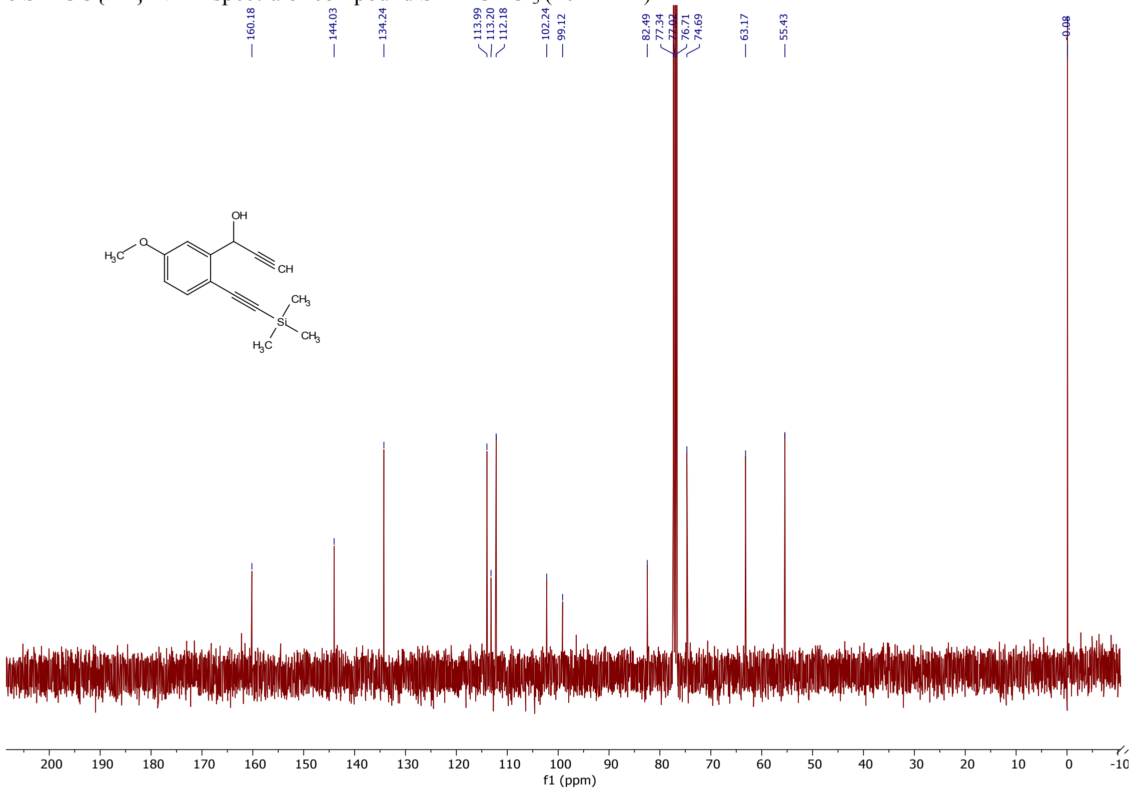

Figure S3 ^{1}H NMR spectra of compound **12** in $CDCl_3$ (400 MHz)

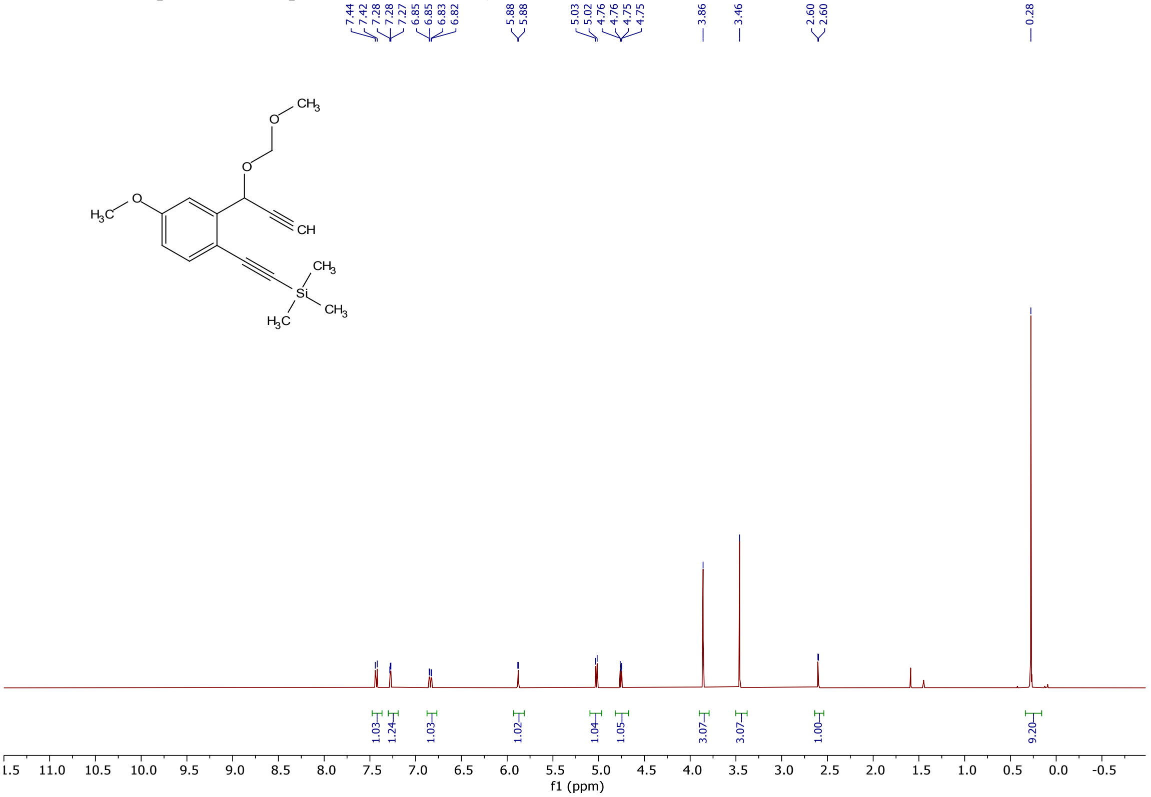

Figure S4 13C{1H} NMR spectra of compound **12** in $CDCl_3$ (101 MHz)

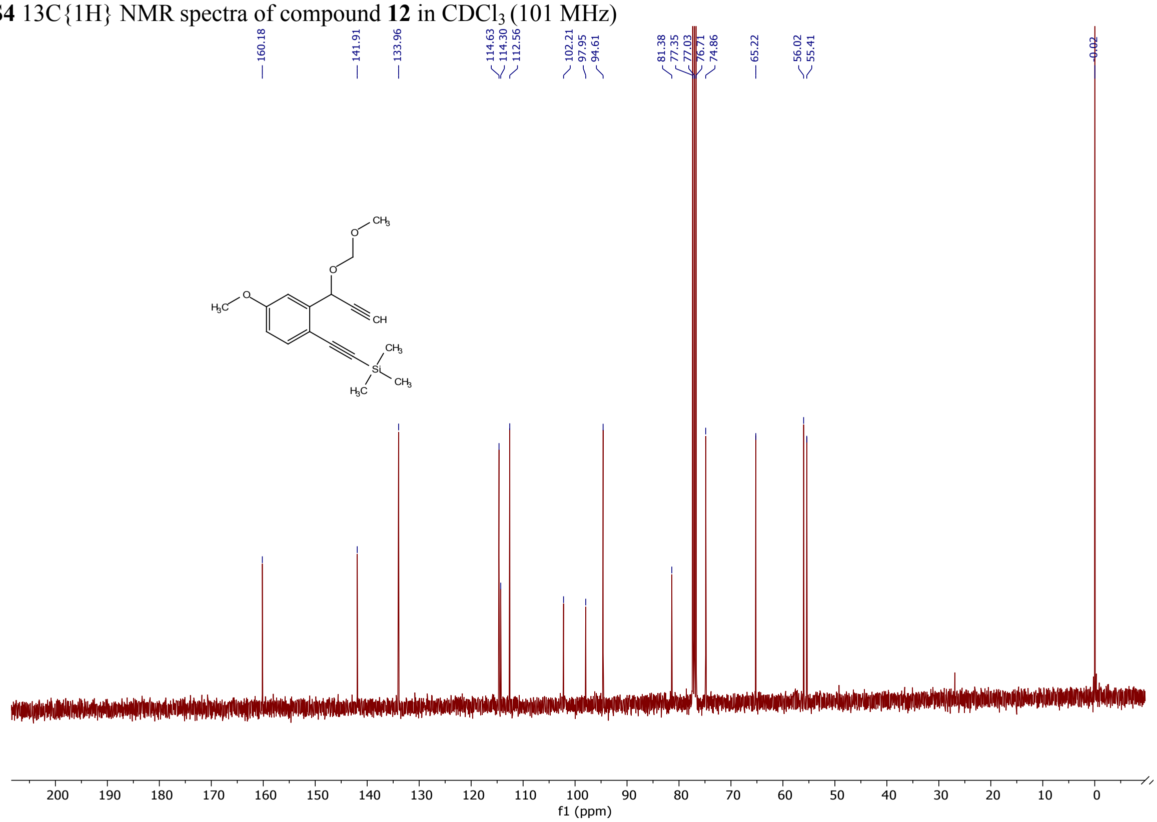

Figure S5 ^{1}H NMR spectra of compound **S2** in $CDCl_3$ (400 MHz)

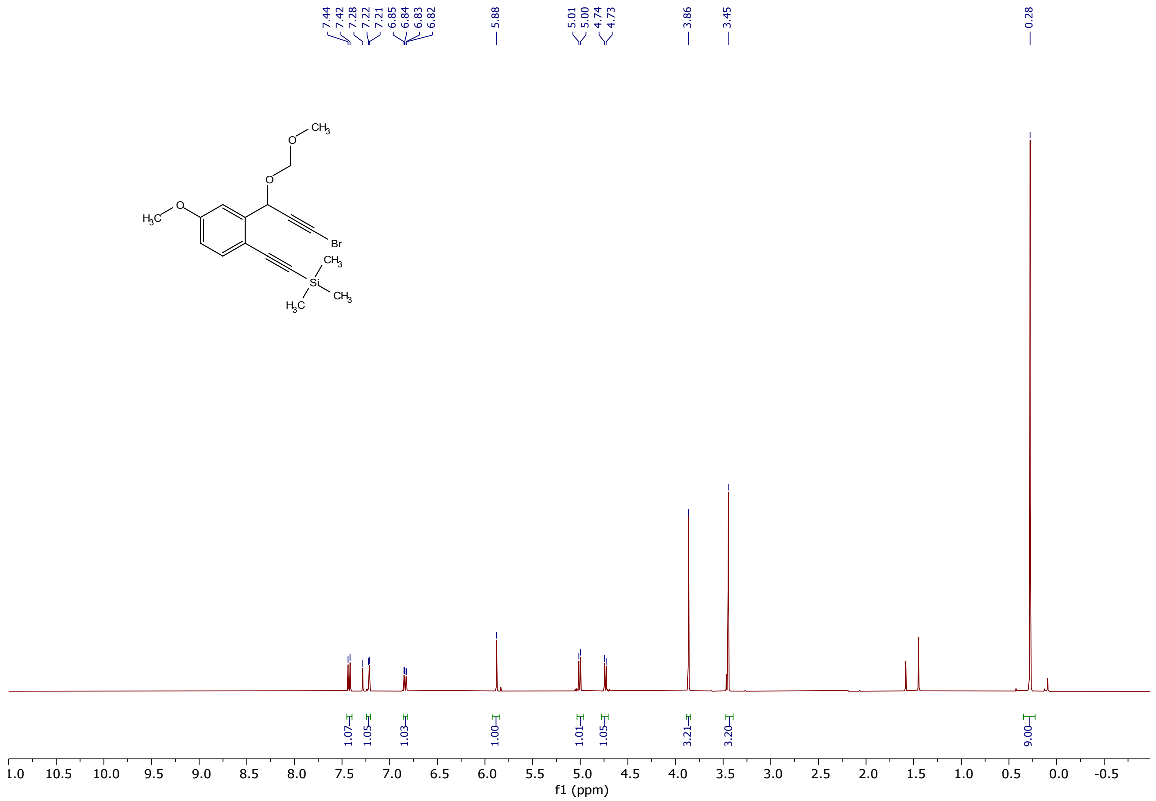

Figure S6 13C{1H} NMR spectra of compound **S2** in $CDCl_3$ (101 MHz)

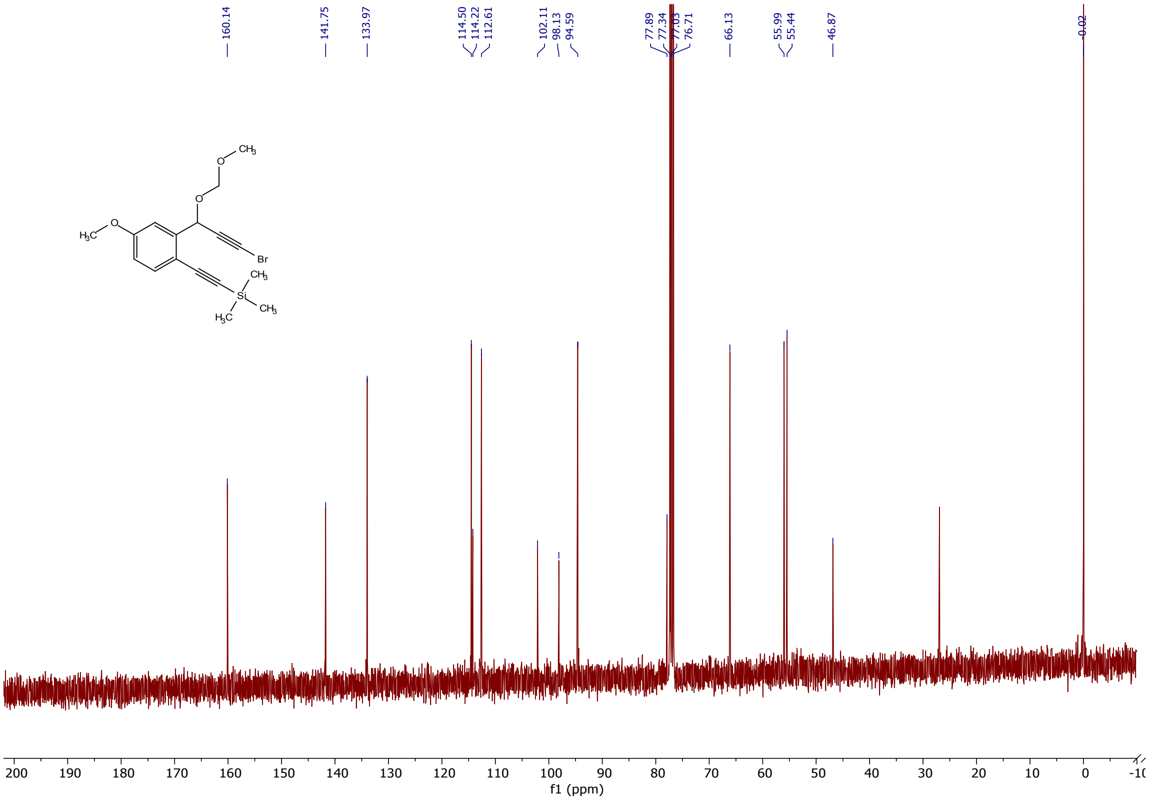

Figure S7 ^{1}H NMR spectra of compound **13** in $CDCl_3$ (400 MHz)

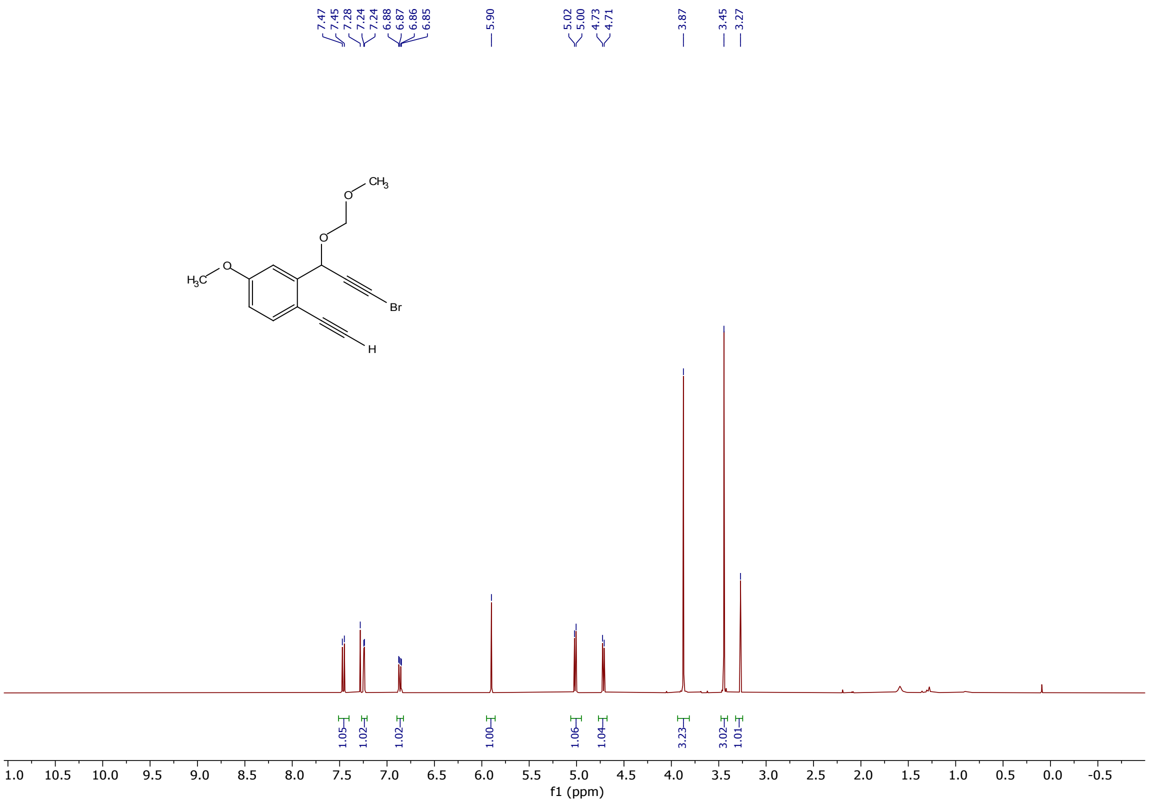

Figure S8 13C{1H} NMR spectra of compound **13** in $CDCl_3$ (101 MHz)

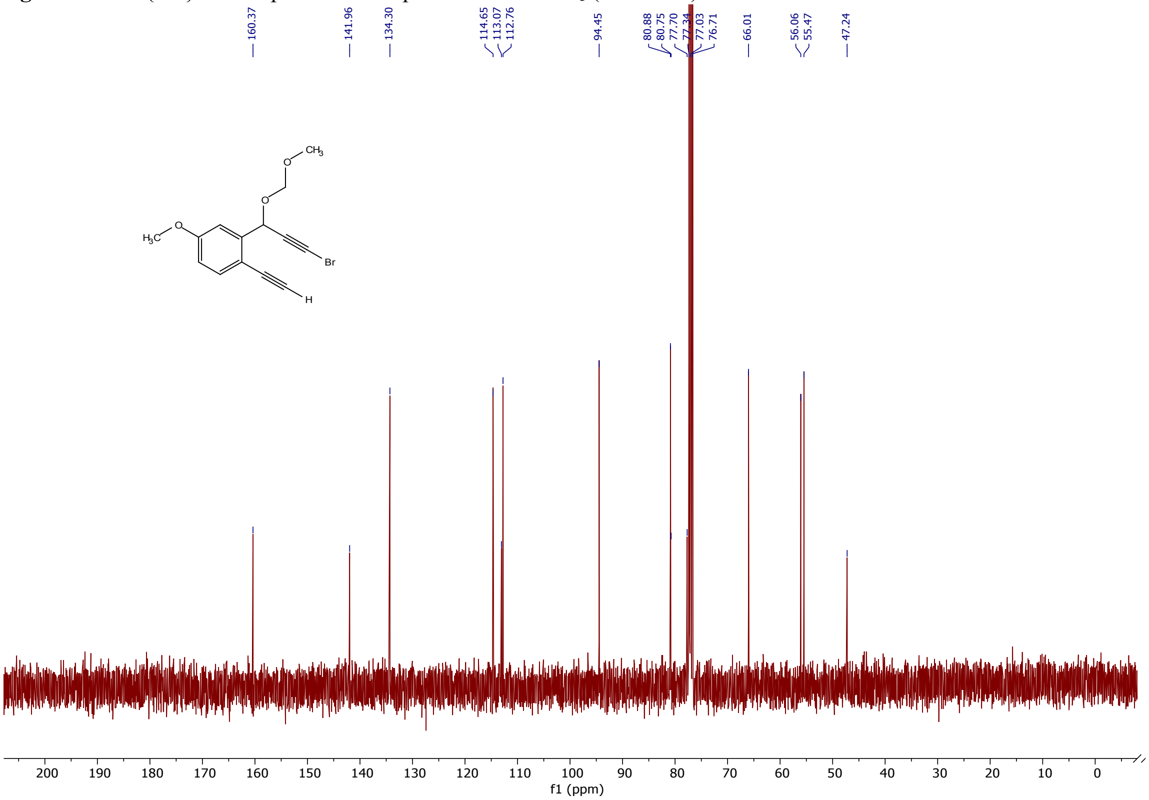

Figure S9 ^{1}H NMR spectra of mixture of compounds **14a** and **14b** in $CDCl_3$ (400 MHz)

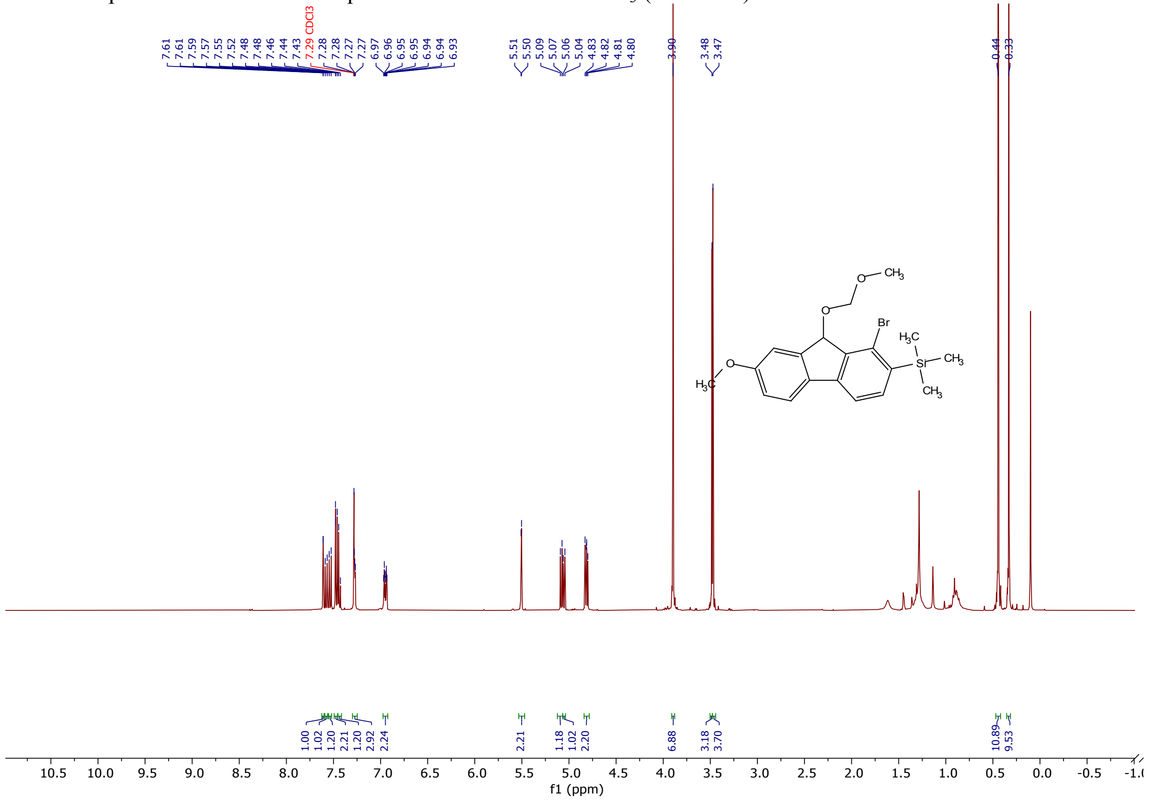

Figure S10 13C{1H} NMR spectra of compound mixture **14a** and **14b** in $CDCl_3$ (101 MHz)

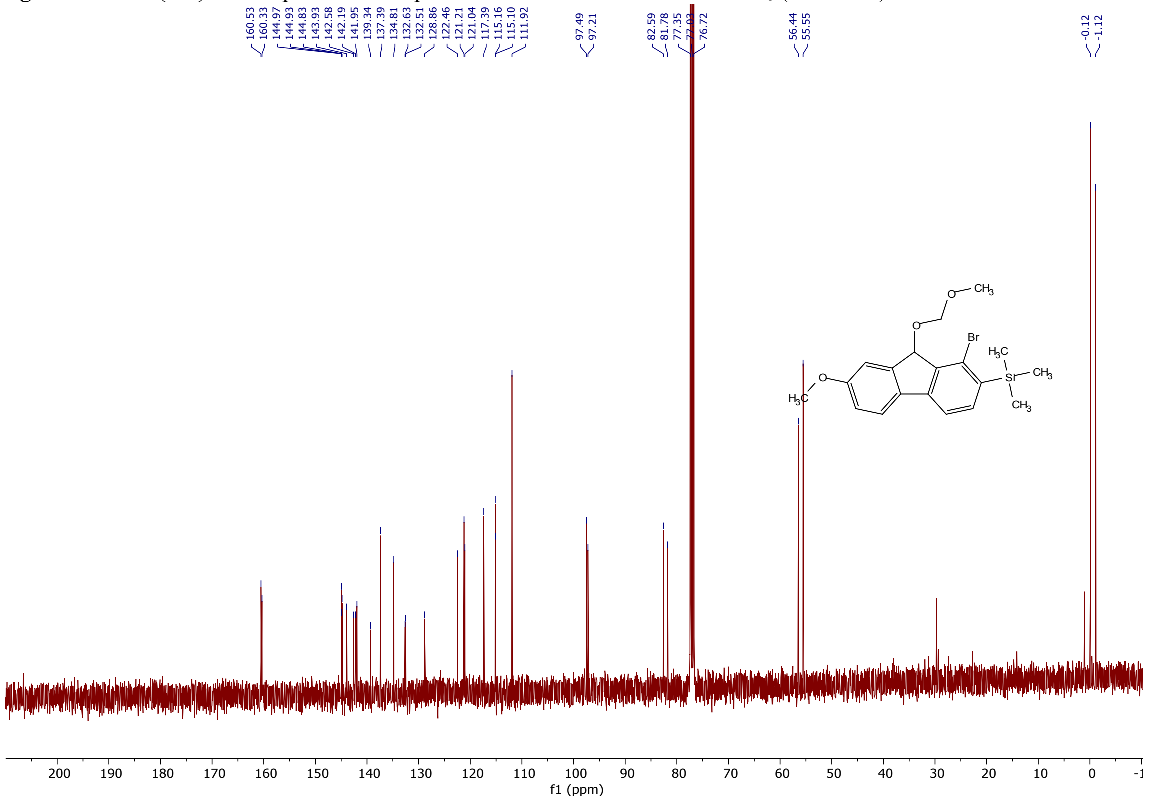

Figure S11 ^{1}H NMR spectra of compound **S3** in $CDCl_3$ (400 MHz)

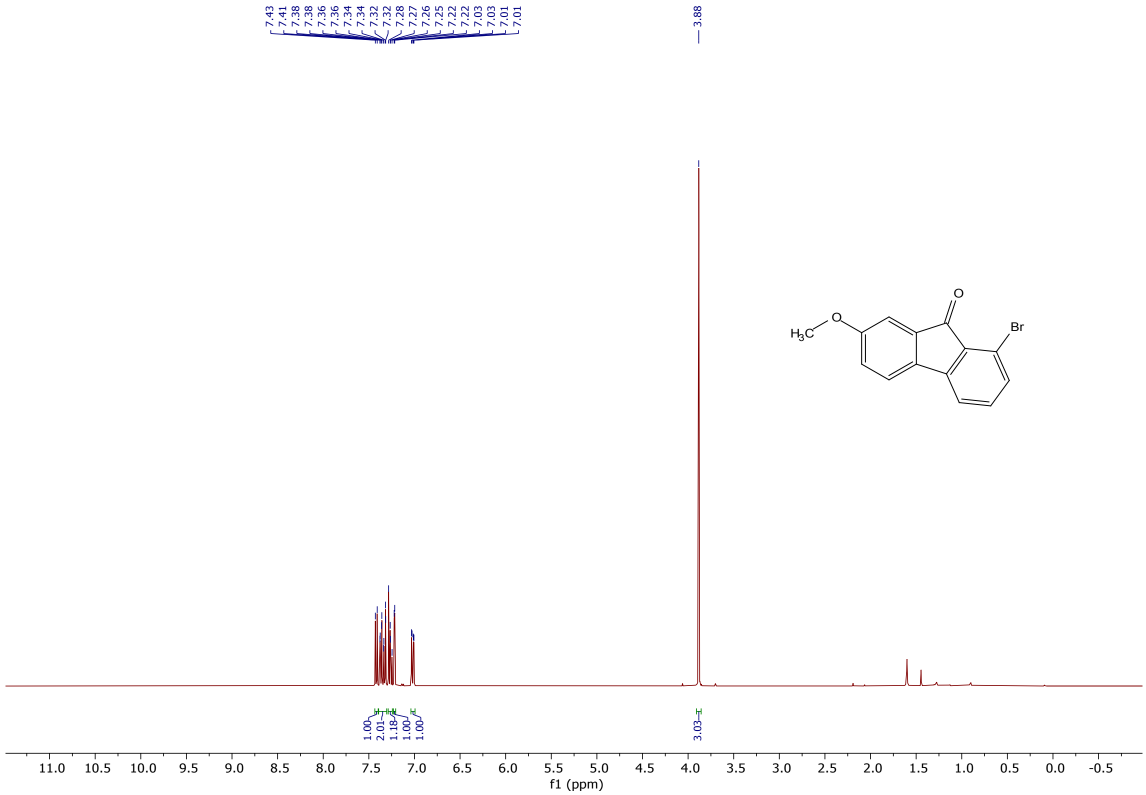

Figure S12 13C{1H} NMR spectra of compound **S3** in $CDCl_3$ (101 MHz)

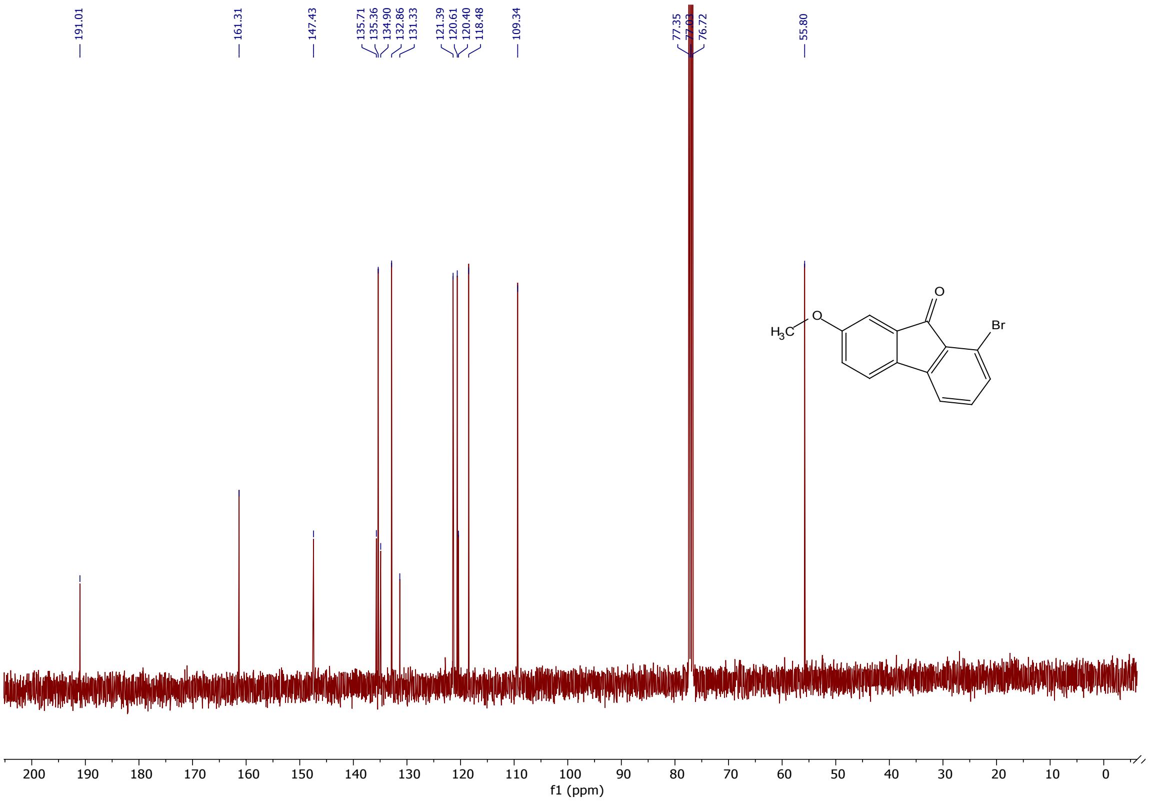

Figure S13 ^{1}H NMR spectra of compound **15** in $CDCl_3$ (400 MHz)

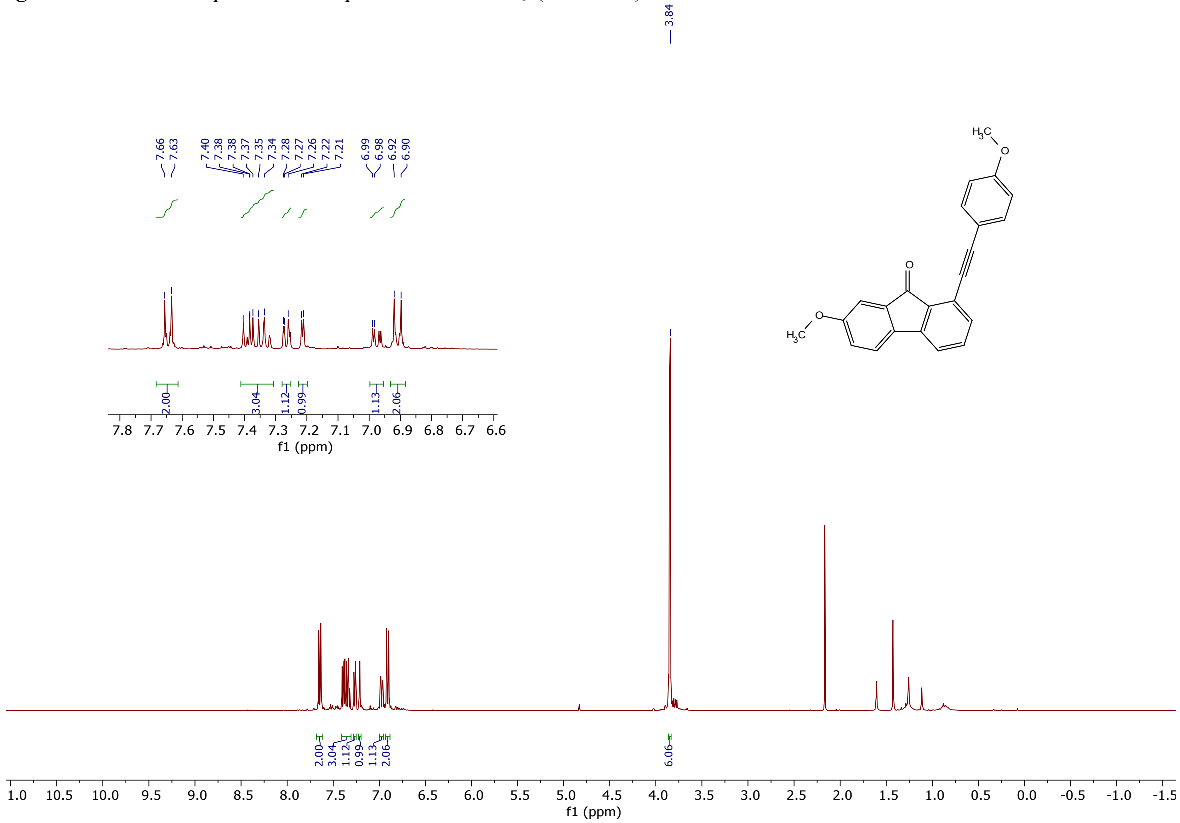

Figure S14 13C{1H} NMR spectra of compound **15** in $CDCl_3$ (101 MHz)

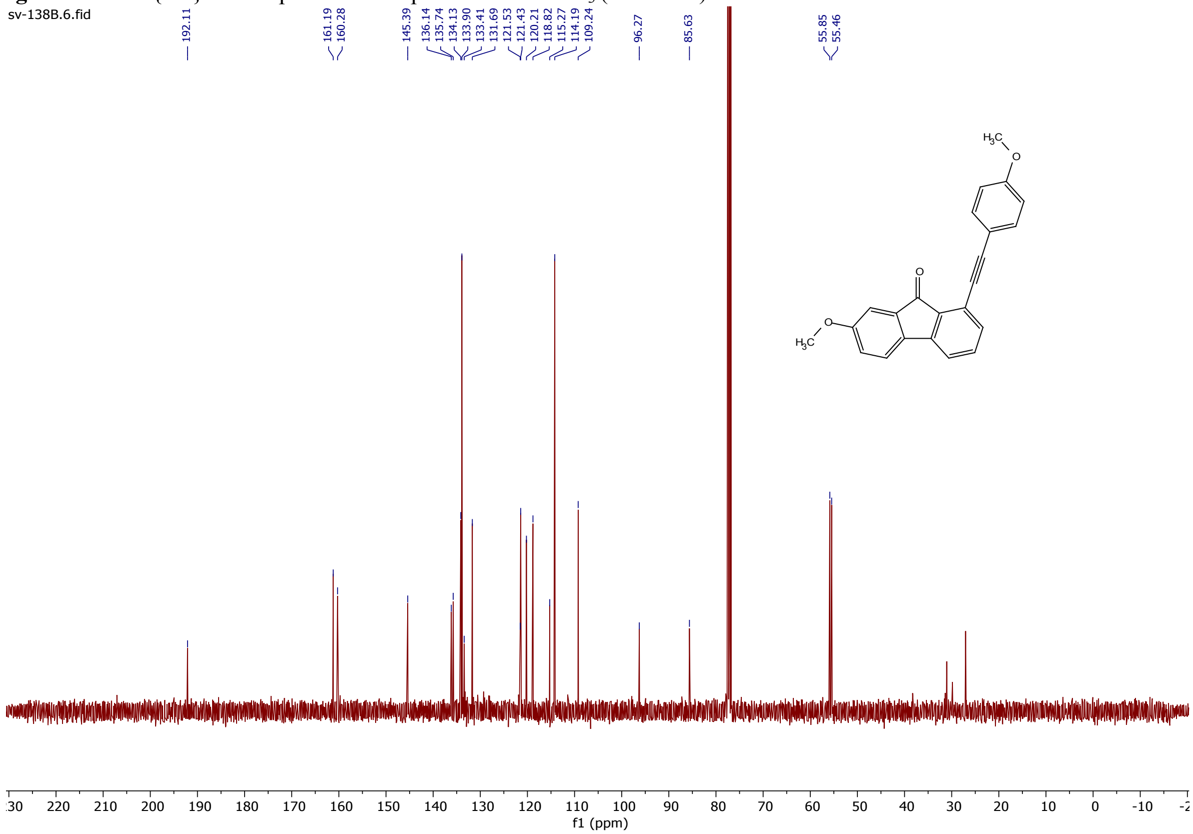

Figure S15 ^{1}H NMR spectra of compound **S4** in $CDCl_3$ (400 MHz)

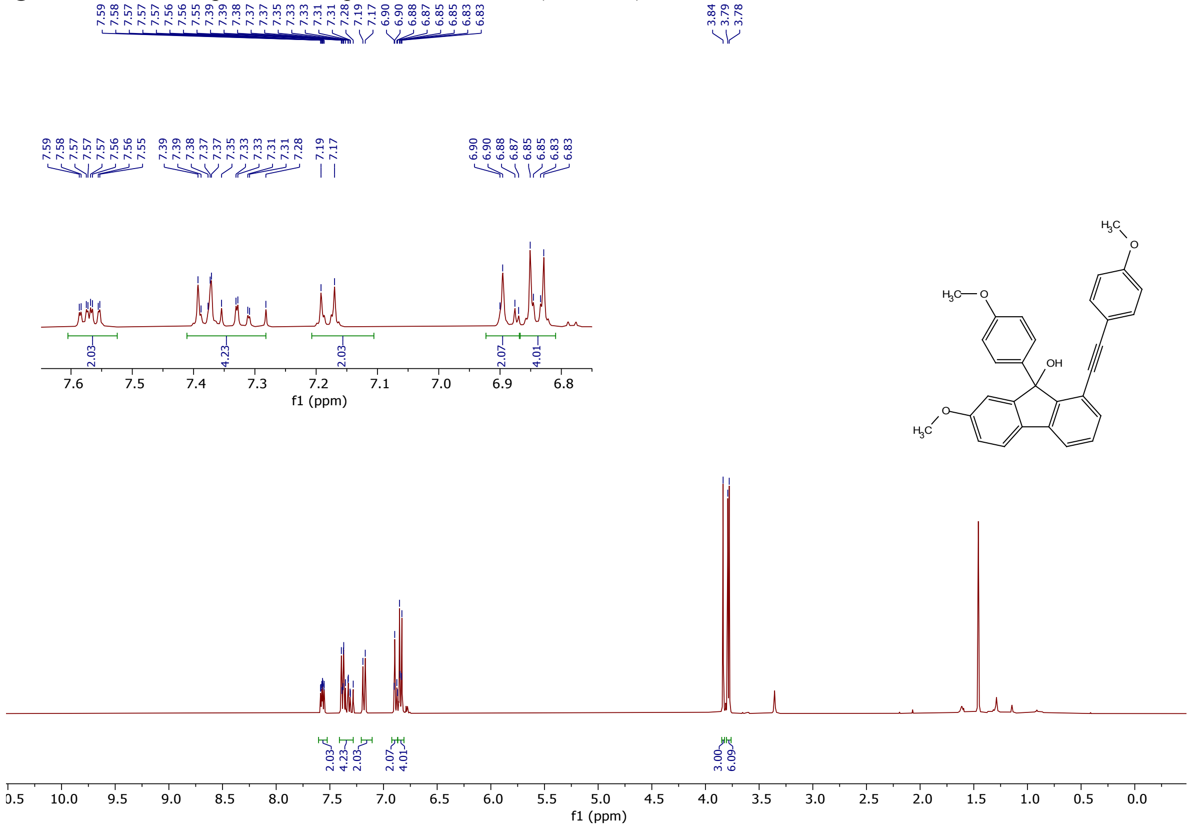

Figure S16 13C{1H} NMR spectra of compound **S4** in $CDCl_3$ (101 MHz)

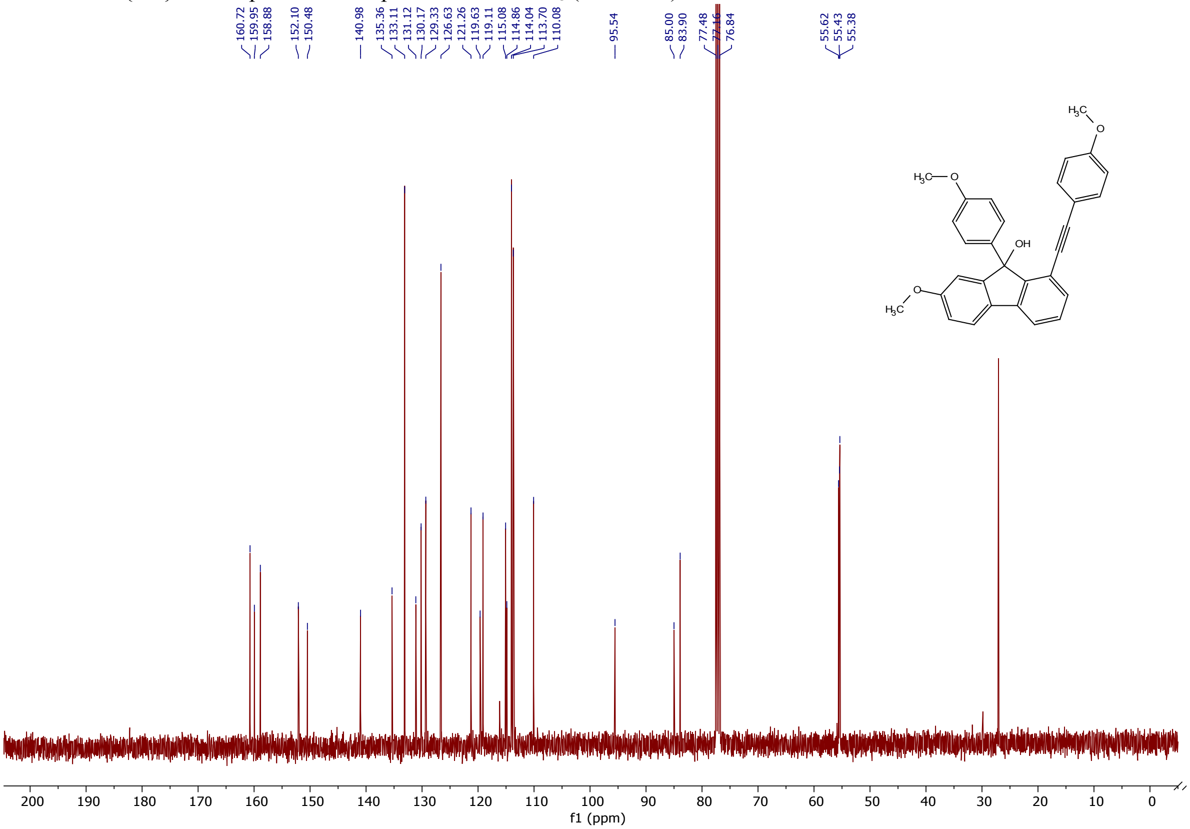

Figure S17 ^{1}H NMR spectra of compound **16** in $CDCl_3$ (400 MHz)

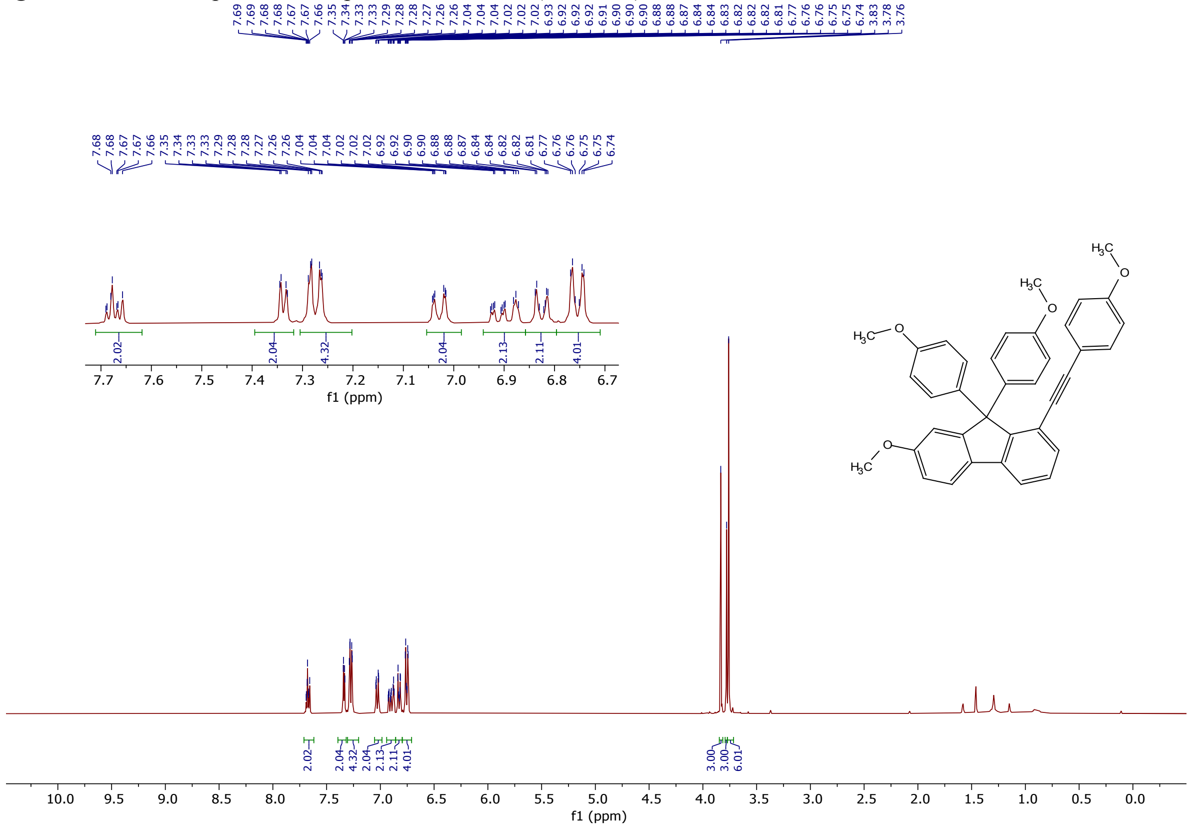

Figure S18 13C{1H} NMR spectra of compound **16** in $CDCl_3$ (101 MHz)

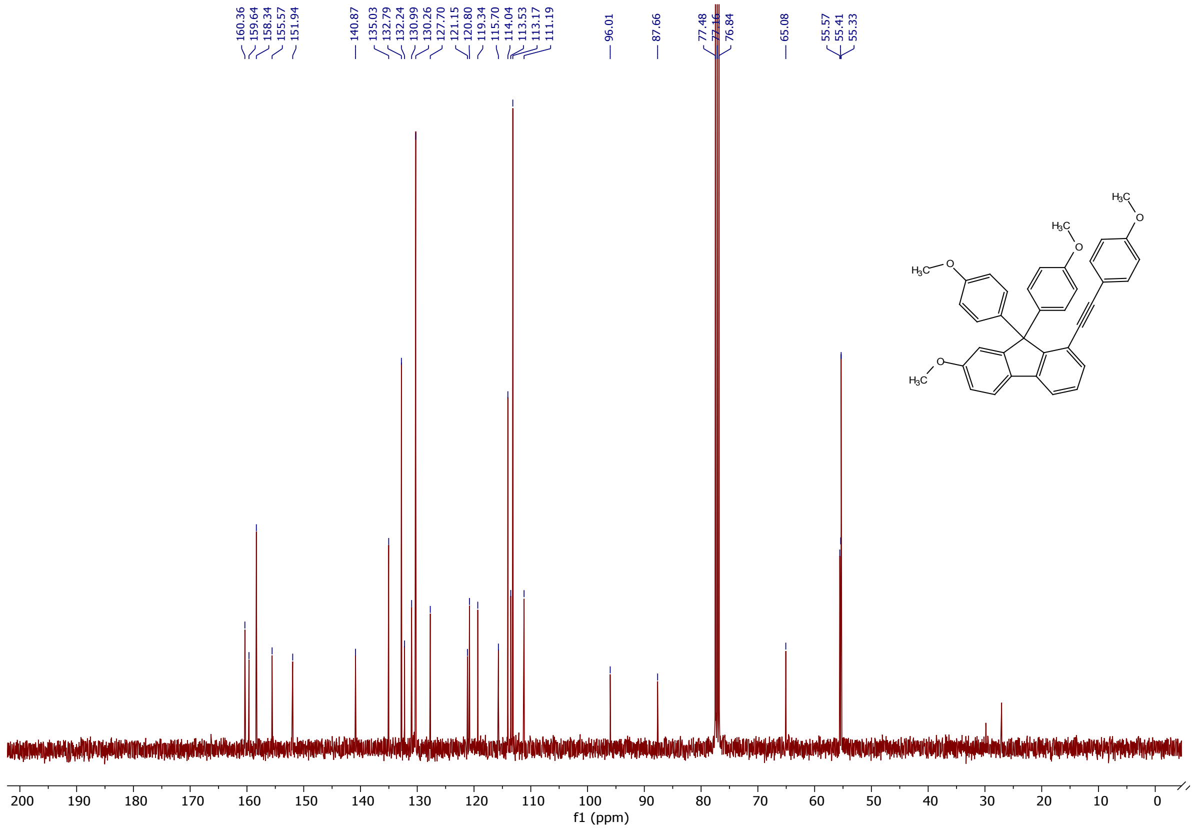

4. References

1. Chinta, B. S.; Baire, B. Formal Total Synthesis of Selaginpulvilin D. *Org. Biomol. Chem.* **2017**, *15* (28), 5908–5911. https://doi.org/10.1039/C7OB00950J.
